# Microwave Wire Interrogation Method Mapping Pressure under High Temperatures

**DOI:** 10.3390/mi9010011

**Published:** 2017-12-29

**Authors:** Xiaoyong Chen, Dan Yan, Yingping Hong, Ting Liang, Jijun Xiong

**Affiliations:** 1 Key Laboratory of Instrumentation Science & Dynamic Measurement, Ministry of Education, North University of China, Taiyuan 030051, China; chenxiaoyong@nuc.edu.cn (X.C.); b1506004@st.nuc.edu.cn (D.Y.); hongyingping@nuc.edu.cn (Y.H.); liangtingnuc@nuc.edu.cn (T.L.); 2 School of Chemical Engineering and Technology, North University of China, Taiyuan 030051, China; 3 National Demonstration Center for Experimental Chemical Engineering Comprehensive Education, North University of China, Taiyuan 030051, China

**Keywords:** pressure sensor, wire interrogation, microwave, high-temperature environment

## Abstract

It is widely accepted that wireless reading for in-situ mapping of pressure under high-temperature environments is the most feasible method, because it is not subject to frequent heterogeneous jointing failures and electrical conduction deteriorating, or even disappearing, under heat load. However, in this article, we successfully demonstrate an in-situ pressure sensor with wire interrogation for high-temperature applications. In this proof-of-concept study of the pressure sensor, we used a microwave resonator as a pressure-sensing component and a microwave transmission line as a pressure characteristic interrogation tunnel. In the sensor, the line and resonator are processed into a monolith, avoiding a heterogeneous jointing failure; further, microwave signal transmission does not depend on electrical conduction, and consequently, the sensor does not suffer from the heat load. We achieve pressure monitoring under 400 °C when employing the sensor simultaneously. Our sensor avoids restrictions that exist in wireless pressure interrogations, such as environmental noise and interference, signal leakage and security, low transfer efficiency, and so on.

## 1. Introduction

In-situ pressure monitoring is highly desirable in a variety of applications, such as aeronautics, vehicle engines, steam turbines in nuclear and thermal-electrical plants, and deep drilling because of its correlation to the safety and reliability, improved performance of components and systems in the applications [1,2]. In an aeronautics engine, its thermal efficiency is the function of pressure inside the burner, and its maximum thermal efficiency needs optimal pressure. Because the safe operation of the engine is closely correlated to pressure, pressure that exceeds its designed value can lead to a compressor surge, consequent flameout, or even serious damage to components in the combustion chamber [3,4]. However, in the aforementioned applications, extreme high temperatures are encountered frequently. For example, the temperature inside a turbojet engine is >1400 °C; turbofan engine, >1700 °C; ramjet engine, >2000 °C; and rocket booster, >3300 °C [1,2,3,4]. Clearly, high temperature is a significant challenge to measuring and controlling instruments, and thus, pressure detection under high temperature has attracted great attention in the global defense, security, aerospace industries, and the scientific community [1].

At present, there are three main pressure-sensing methods for high-temperature environments. One method is based on active pressure-sensitive devices such as field effect transistor [5,6,7], piezoelectric active sensor [8], P-N junction based piezoresistive sensor [9], and so on. The electronic effect of active devices fade, even disappear, as temperature increases; therefore, active devices usually work at temperatures below 600 °C [3,6,10,11,12], far lower than the requirement of aeronautical applications. The second method employs passive pressure measuring devices with electronic interconnection [13,14,15,16,17]. In these devices, piezo resistive and capacitive pressure sensors are typical. However, both have low operating temperatures because of heterogeneous wire interrogation failures, electrical conduction performance deterioration under high temperatures. For example, the working temperatures of Liang et al.’s [15] silicon on insulator (SOI)-based, resistive, wire interconnected pressure sensor are below 350 °C; Fricke et al.’s [16] sapphire-based sensor, below 440 °C; Yang’s [18] SiC-based sensor, below 450 °C; Young et al.’s [19] 3C-SiC-based, captive wire interconnected pressure sensor, below 350 °C; Marsi et al.’s [17] 3C-SiC-based sensor, below 500 °C; and Chen et. al.’s [20] SiC-based sensor below 600 °C. The last method utilizes passive, wireless pressure sensor. The passive wireless sensors are believed as the most promising and practical for high-temperature applications because there is no need to power support and wire interrogation, and therefore, no trouble in periodic battery renewal or electronic signal delivery failure. Consequently they have been investigated and applied intensely, such as pressure sensing [2,3,21,22,23,24,25,26,27,28], temperature reading [29,30,31,32] and strain monitoring [33,34,35], crack detecting [36], structural health monitoring [37], and so on. LiDAR (Light Detection and Ranging), SAW (Surface Acoustic Wave), LC (Inductance and Capacitance) inductive, and microwave wireless pressure solutions are representative. Because LiDAR cannot work in opaque environment and SAW is limited by Curie point temperature of piezoelectric materials, LC inductive and microwave non-contact pressure transducers attract more attention. Allen et al. [38,39]. and Yang [18] mapped pressure profiles at 450 °C using LC resonant circuits, while Xiong et al. and Tan et al. [21,22,40] achieved pressure detection at 800 °C with a passive wireless LC-resonator structure. Jatlaoui et al. proposed microwave transduction wireless passive pressure sensor and investigated the pressure-sensing performance of the sensor at ambient temperature [41,42], Senior et al. also measured ambient pressure based substrate integrated waveguide resonator [43], while Gong et al. [3] exploited a passive wireless pressure sensor based on microwave transduction, working at 800 °C.

However, new issues emerge when using passive-wireless pressure sensors: (1) environmental noise interferes and submerges working signals; and (2) under high-temperature conditions, coupling energy is heavy dissipated, resulting in decreased or inoperable wireless interrogation distance and pressure detecting sensitiveness [30,32]. The noise interfering and energy dissipation limit wireless pressure sensor applications, especially inside metal vessel. As a result, new pressure monitoring solutions, such as improving Q techniques and time-gating interrogation [30,32], are developed for high-temperature environments. Boccard et al. [30] used dielectric resonator to improve Q factor (>670 at 700 °C) and realizing temperature information acquirement up to 700 °C, while Huang et.al. [32] developed time-gating interrogation technique for diminishing noise effect and demonstrated temperature sensing up to 280 °C. These works enlighten us to do Q improvement and noise suppression in pressure sensors. The microwave wire interrogation method may be a good alternative which combines Q enhancement with noise elimination due to low path loss and good metal shielding. 

In this article, we develop a new solution for measuring gas pressure in high-temperature environments based on wired microwave transducers. In this solution, we employ a microwave resonator as the pressure-sensitive element and a microwave transmission line as the pressure-signal carrier. The solution utilizes electromagnetic wave propagation in various dielectrics and hollow transmission lines which casts off the electron-current dependence of signal interrogation and thus avoids signal dissipation, depletion, and avalanche at high temperatures [44]. In the microwave transmission line, pressure signals are delivered in self-isolated conditions; therefore, disturbance from environmental noise is minimized. At the same time, energy can be transferred to pressure-sensitive elements by transmission lines, and therefore, energy dissipation, further interrogation distance, can be improved.

In this article, we demonstrate the suitability of the microwave wire-interrogation method for sensing pressure under high temperatures. We present the working principles of the system architecture in the section “Pressure Detection Principle and System Description” and the pressure detection results in the section “Experimental Results & Discussion”.

## 2. Pressure Detection Principle and System Description

**Figure 1:**
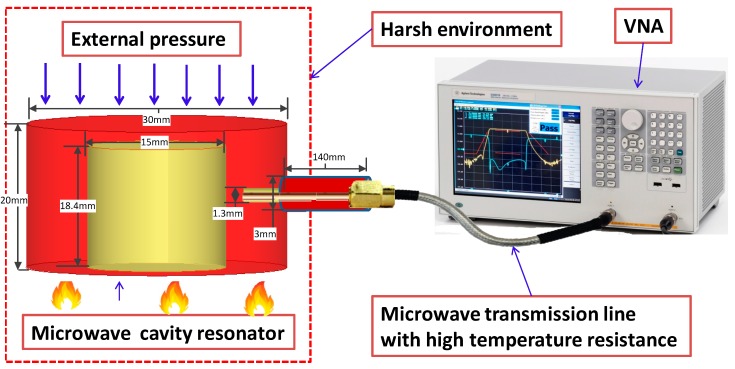
Schematic of the wire interrogation, in-situ pressure-monitoring configuration used in high-temperature environments.

The schematic of the developed pressure sensor and wire interrogation mechanism is illustrated in Figure 1. The sensor is composed of an evanescent-mode resonator and a microwave transmission line with high-temperature resistance. The evanescent-mode resonator is used as a pressure-signal capturing cell, which is also applied extensively in the flow [45] and temperature [46] detection fields. The resonator is characterized by high Q factor, high sensitivity, small size, low work frequency, and ease of design. The resonator is based on a cut-off coaxial waveguide with two shorted metal plates; however, the central cylindrical post inside the resonator is shorter than the height of the resonator cavity and thus causes the parallel-plate capacitance $c_{p}$ between the post and the resonator cap (top-shorted metal plate). Via the resonator, the pressure variable is extracted from its resonating frequency (*fr*), which changes with its volume deformation.

Since the design of evanescent-mode resonator is widely detailed in [3], we briefly summarize it here. The *fr* of the resonator is determined by [3,45].

(1) $$f_{r}=\frac{1}{2\pi \sqrt{L(c_{p}+c_{l})}}$$

where *L* is the equivalent inductance of the resonator, and $c_{l}$ is the fringing capacitance between the resonator cap and the central post, which is far less than $c_{p}$. The simplified Equation (1) is frequently written as

(2) $$f_{r}=\frac{1}{2\pi \sqrt{Lc_{p}}}$$

where $c_{p}$ can be approximately calculated as

(3) $$c_{p}=\frac{\varepsilon_{0}A}{d}$$

where *A* is the top surface area of the post inside the resonator, and *d* is the spacing between the resonator cap and the post.

The pressure detecting mechanism of the resonator-based pressure sensor is extracting the pressure variable from the resonating frequency (*fr*) due to the *fr* change with the volume deformation. While external pressure is exerted on the resonator, the spacing *d* reduces and further increases the $c_{p}$ from Equation (3), and *fr* shifts down based on Equation (2). Obviously, the *fr* of the resonator is correlated to external pressure, and the mathematic connection of *fr* with an external pressure can be built. As a result, the external pressure is the inverse problem of the *fr* function of the resonator.

## 3. Methods 

**Figure 2:**
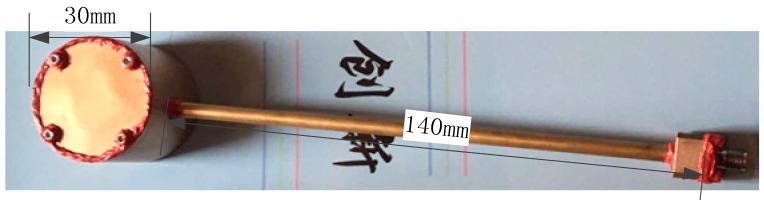
Demo of the microwave wire interrogation, high-temperature pressure sensor.

In order to prove the effectiveness and flexibility of the proposed pressure sensor, we designed and fabricated a demo device (Figure 2).

The demo microwave wire interconnected pressure sensors composed of a coaxial-like cavity resonator and microwave waveguide. The sensor works theoretically at 2.56 GHz of central frequency via a high-frequency structure simulation (HFSS) and a practical work frequency of 2.68 GHz. The cavity resonator is made of copper (brass H62, Kunshan AmptonFine Materials Ltd., Kunshan, Jiangsu, China), with the cavity size of *Φ =* 30 mm × 20 mm, and the central post size, *Φ =* 15 mm × 18.4 mm. The waveguide is a non-standard, domestic coaxial transmission line that consists of an external copper tube with a bore size of *Φ =* 3 mm, an inner copper bar with an outside diameter of *Φ =* 1.3 mm. The waveguide has a length of 14 cm. One end of the waveguide is weld to the cavity, and the other end is terminated with an SMA male connector. The cavity and waveguide are sealed with high temperature silicone adhesive (LOCTITE^®^ 596™, Henkel LOCTITE Asia-Pacific, Yantai, Shandong, China).

**Figure 3:**
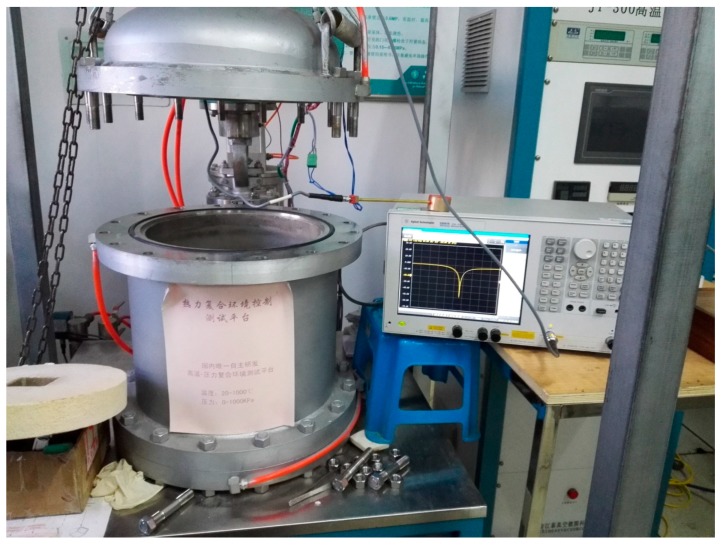
Experimental validation measurement setup.

The pressure sensing performance of our sensor is characterized via a homemade thermal/pressure composite measurement platform (Figure 3), which can operate up to 1000 °C and 1 MPa. The measurement platform is a full metal alloy structure with good thermal resistance. A heater at the bottom of the platform regulates the test temperature, and a gas pressure valve that connects a nitrogen gas cylinder controls the test pressure. At the beginning, our sensor is positioned on the heater and the platform is closed, then the air in the platform is replaced by nitrogen gas. The sensor is connected to a network analyzer (E5061B, Agilent, Santa Clara, CA, USA). The heater is heated to the aimed temperature with 10 °C/min. After 10 min at the temperature, nitrogen gas is pumped into the platform until the desired pressure. The network analyzer transmitted a sweep signal with a certain bandwidth to our sensor, and then records the reflection characteristics (*S*_11_) of the sensor. The minimum of *S*_11_ is corresponding to the resonating frequency of the sensor. The resonating frequency changes with external pressure. For demonstrating the work mechanism of the wire interrogation sensor, here the response of the sensor under 50 kPa, 100 kPa and 150 kPa, respectively, were tested.

## 4. Experimental Results & Discussion

**Figure 4:**
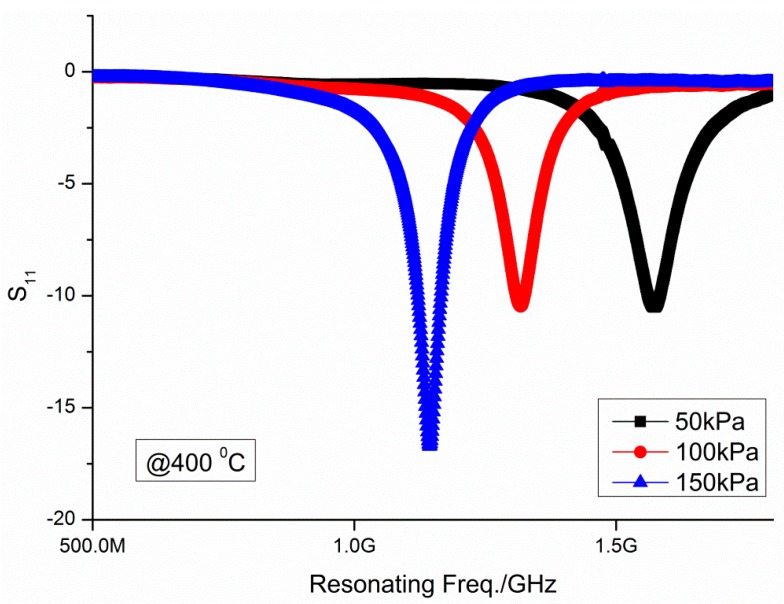
Scatter parameter *S*_11_ vs. frequency plots of the developed sensor.

Figure 4 illustrates the scatter parameter *S*_11_ vs. frequency plots of the developed sensor at 400 °C. All these plots have concave profiles with a single “valley”. According to the electromagnetic wave theory, the “valley” denotes the resonating frequency of the resonator. When the gas pressure rises at any temperature, the “valley” shifts to a lower frequency, complying with the principal rules. For conciseness, the other pressure data under 100 °C, 200 °C and 300 °C, are not shown.

**Figure 5:**
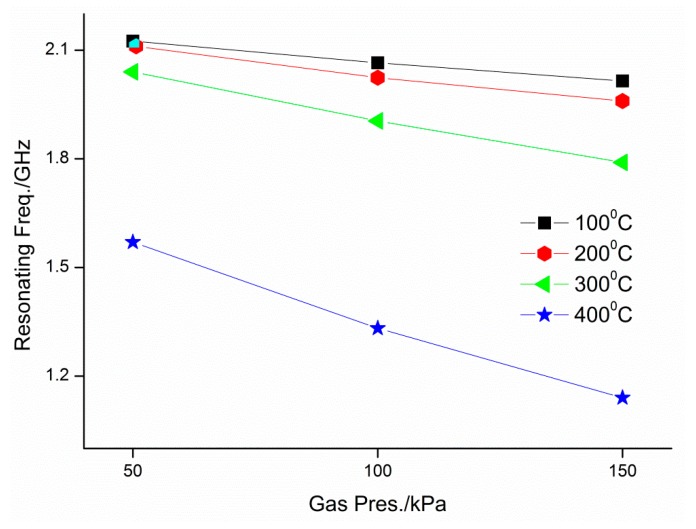
Measured resonant frequency of the developed sensor vs. the applied pressure at each temperature.

Resonant frequency vs. pressure at various temperatures is plotted in Figure 5. The figure shows that resonant frequency decreases with increasing gas pressure in a quasi-linear manner because of the diminished spacing dimensions. For example, the resonant frequency is reduced from 1.57 GHz to 1.14 GHz when the external pressure increases from 50 KPa to 150 KPa at 400 °C. At higher experimental temperature, pressure change caused by a pressure is larger, compared to that of lower temperature. It may be that copper diaphragm softens with heat. As a result, the same pressure will induce larger deformation under elevated temperature, further larger frequencies shift down in our pressure actuator. In addition, when the temperature increases under a pressure, the resonant frequencies of the developed pressure sensor drop owing to thermal expansion of copper materials, trapped air swell in the sensor with heat, or residual stress inside the adhesive and the copper material, especially in weld sector [3,28]. 

Some pressure sensors for high temperature applications are summarized in Table 1. It can be seen that much higher sensor sensitivity is achieved by using microwave resonators, compared with other pressure sensors based on LC resonators, while evanescent-mode resonators far exceed non-evanescent-mode resonator. Further, our pressure sensing solution with wire interconnection has highest sensitivity. Wire interconnection interrogating has lower path loss, and thus higher Q factors and higher sensor sensitivity. Due to using low thermal resistance materials, our sensor work temperature is lower than reported results.

## 5. Conclusions 

In this article, we successfully demonstrated a microwave wire-interrogated pressure sensor for use in high-temperatures up to 400 °C. The proposed sensor can avoid environmental disturbance and compensate for energy dissipation in high temperatures owing to the isolated signal and power delivery system. In addition, further studies will be carried out on the resonator and transmission line design; on impedance matching to reduce electromagnetic reflection and loss; and on improving the selection of the thermal resistance materials to enhance applicability above 400 °C, 500 °C, and even 1000 °C. In principal, this blueprint could be achieved because Guo et al. employed a wire interconnection microwave resonator for complex dielectric properties up to 2000 °C [47].

## Figures and Tables

**Table 1:** Characteristics of different pressure sensors for harsh-environment applications.

Cases	Working Principle	Sensors Materials	Highest Working Temperature (°C)	Highest Working Pressure (kPa)	Sensitivity (MHz/kPa)
[38]	LC Resonator	LTCC and silver	400	700	0.00141
[27]	LC Resonator	LTCC and silver	600	360	0.00344
[26]	LC Resonator	HTCC and platinum	600	300	0.0000086
[3] ^a^	Evanescent-mode resonator	PDC and platinum	800	52.6	3.6
[28]	Re-entrant resonator	HTCC and silver	800	120	0.73125
This work	Evanescent-mode resonator	Copper	400	150	4.3

^a^ The pressure applied to the sensor is through a dielectric rod not ambient gas. LC—inductance and capacitance; LTCC—Low Temperature Co-fired Ceramic; HTCC—High Temperature Co-fired Ceramic; PDC—polymer derived ceramic
